# Antioxidant and Anti-Inflammatory Effects of Different Zinc Sources on Diquat-Induced Oxidant Stress in a Piglet Model

**DOI:** 10.1155/2020/3464068

**Published:** 2020-03-21

**Authors:** Jieping Guo, Liuqin He, Tiejun Li, Jie Yin, Yulong Yin, Guiping Guan

**Affiliations:** ^1^College of Animal Science and Technology, Hunan Agricultural University, Changsha, 410128 Hunan, China; ^2^Scientific Observing and Experimental Station of Animal Nutrition and Feed Science in South-Central, Ministry of Agriculture, Hunan Provincial Engineering Research Center of Healthy Livestock, Key Laboratory of Agro-ecological Processes in Subtropical Region, Institute of Subtropical Agriculture, Chinese Academy of Sciences, Changsha, Hunan 410125, China; ^3^Hunan International Joint Laboratory of Animal Intestinal Ecology and Health, Laboratory of Animal Nutrition and Human Health, College of Life Sciences, Hunan Normal University, Changsha 410081, China

## Abstract

Zinc (Zn) plays a crucial role in reducing oxidative stress and diarrhea in postweanling piglets. This study is aimed at comparing the effects of zinc chelate of 2-hydroxy-4 methyl-thio butanoic acid (HMZn) and ZnSO_4_ on the oxidative stress in weaned piglets. A total of 32 piglets were randomly divided into 4 treatments: CON: basal diet+80 mg/kg Zn as ZnSO_4_; DIQ: basal diet+80 mg/kg Zn as ZnSO_4_; HMZn: basal diet+200 mg/kg Zn as HMZn; and ZnSO_4_: basal diet+200 mg/kg Zn as ZnSO_4_. On day 15, the DIQ, HMZn, and ZnSO_4_ groups were injected intraperitoneally with diquat except for the CON group. The trial lasted 21 days. The results showed that zinc sources did not influence the growth performance during the first 14 days. But HMZn increased activities of superoxide dismutase (SOD), glutathione peroxidase (GPX), and total antioxidant capacity (T-AOC) in serum (*P* < 0.05). After diquat injection, the fecal score was decreased in the HMZn group. Both HMZn and ZnSO_4_ increased the activities of GPX and T-AOC in serum and the relative mRNA expressions of hepatic and renal Nrf2, SOD1, and GPX compared with the DIQ group (*P* < 0.05). Moreover, the relative mRNA expression of inflammatory factors in the small intestine, liver, and kidney was downregulated; the phosphorylation of NF-*κ*B protein was inhibited in the HMZn group compared with the DIQ and ZnSO_4_ groups (*P* < 0.05). In general, HMZn showed notable advantage over ZnSO_4_ in reducing diarrhea and improving antioxidant and anti-inflammatory ability in piglets challenged with diquat.

## 1. Introduction

Zinc (Zn), as an essential constituent of more than 200 enzymes involving antioxidant enzymes, plays important roles in their biochemical and pathophysiological functions [1]. An increasing amount of evidence suggests that Zn is one of the redox-active trace minerals and participates in the modulation of intracellular and extracellular redox state [2]. Zinc supplementation was demonstrated to significantly improve growth performance, antioxidant ability, and immune function of weanling pigs [3]. Although weaning piglets are highly susceptible to weaned syndrome, including growth retardation, diarrhea, metabolic disorders, and even death, dietary Zn supplementation could improve growth performance in the weaning stress conditions [4–7]. Many forms of inorganic Zn (i.e., ZnSO_4_, ZnO) have been used in weanling piglets' diets to improve growth performance in the last decades; for example, a pharmacological level of ZnO (1500~3000 mg Zn/kg diet) is commonly used to prevent diarrhea and deemed as an alternative for antibiotics in weaning piglets [8–10]. While there is certain disputation on bioavailability of Zn from different sources, an increasing number of reports show that megadose of in-feed inorganic Zn with low absorption efficiency has been the source of environmental pollution [4, 11, 12].

HMZn, chelated by the hydroxy analogue of methionine (HMA) and Zn, is chemically stable and could be decomposed to HMA and Zn in the acid gastrointestinal tract. Diffusion and monocarboxylate transporters are the main transport pathways for HMA absorption; thus, HMA avoids competitions from amino acid transporter-dependent amino acids (Martin-Venegas et al. 2007; Fang et al. 2010b). The absorbed HMA is subsequently converted to methionine and acts as an endogenous antioxidant in cells. Some recent reports indicate that organic Zn in pigs is more conducive to mediate the adaptive response to piglets' oxidative stress compared with inorganic Zn [13, 14]. However, the effect of HMZn on regulating antioxidant and anti-inflammatory ability has not been well studied, especially on comparative effect of HMZn and ZnSO_4_ in piglet nursery diets and evaluated potential benefits of organic HMZn on the intestinal antioxidant capacity of piglets in oxidative stress conditions. Considering its stable structure and potential supply of methionine, we hypothesized that HMZn might be advanced in antioxidative and anti-inflammatory capacity compared with ZnSO_4_. To test the hypothesis, diquat-challenged piglets were used as oxidative stress models to investigate effects of HMZn and ZnSO_4_ on antioxidant capacity and anti-inflammatory gene expression in the intestine.

## 2. Materials and Methods

### 2.1. Animal Experiment

This study was approved by the Laboratory Animal Welfare Commission of the Institute of Subtropical Agriculture, Chinese Academy of Sciences.

A total of 32 (35-day-old) health piglets (Duroc × Landrace × Yorkshire, 9.41 ± 0.11 kg BW) were allotted randomly to the following treatments: (1) control group (C): basal diet+80 mg/kg Zn as ZnSO_4_; (2) negative control (NC): basal diet+diquat+80 mg/kg Zn as ZnSO_4_; (3) HMZn group (HMZn): basal diet+diquat+200 mg/kg Zn as HMZn; and (4) ZnSO_4_ group (ZnSO_4_): basal diet+diquat+200 mg/kg Zn as ZnSO_4_. Basal diet was formulated to meet the nutrient requirements recommended by the National Research Council (2012) and is shown in Table 1. Animals were housed in individual pens and had free access to water and feed. On day 15, the 2 to 4 groups were injected intraperitoneally (i.p.) with 10 mg/kg BW diquat (Diquat Dibromide Monohydrate, PS365; Sigma Co.) to induce oxidative stress, while the control group was intraperitoneally injected with the sterile saline. On day 21, all pigs were anaesthetized according to our previous methods; then, the blood and small intestine were sampled [15].

### 2.2. The Determination of Growth Performance and Diarrhea Index

The daily feed intake and the body weight per piglet were recorded during the whole experimental period in order to calculate the average daily gain (ADG), average daily feed intake (ADFI), and food conversion rate (FCR). The fecal consistency was daily observed and scored for individual piglets according to the following criterion. Diarrhea index was calculated according to a previous study [16].

### 2.3. Intestinal Histomorphology

The duodenum, jejunum, ileum, and colon samples were fixed with neutral formalin solution and then embedded in paraffin. After being cut into approximately 5 *μ*m thicknesses, the paraffin sections were stained with hematoxylin and eosin. The intestinal villus height and crypt depth were measured using a light microscope equipped with a calibrated eyepiece graticule (BioScan Optimetric, BioScan Inc., Edmonds, WA, USA) [17]. Quantitative analysis of the digitally acquired images was performed by ImageJ software. The ratio of villi and crypt was calculated.

### 2.4. The Determination of Serum Antioxidant Enzymes

On days 7, 14, 17, and 21, blood samples were collected from jugular vein puncture, and the serum was obtained after centrifugation (3000 × g, 10 min, 4°C). The prepared serum samples were stored at −20°C for further analysis. The activities of total superoxide dismutase (T-SOD), glutathione peroxidase (GPX), total antioxidant capacity (T-AOC), catalase [18], and concentrations of malondialdehyde (MDA) were determined by commercial ELISA kits according to the instruction (Nanjing Jiancheng Bioengineering Institute, China).

### 2.5. Quantitative Real-Time PCR

Total RNA of the liver, kidney, and intestinal samples was isolated using TRIzol reagent (Invitrogen, China), and the quality of RNA was further checked on 1% agarose gel electrophoresis. Then, the cDNA was synthesized by a PrimeScript™ RT Reagent Kit (Takara, China) according to the instructions. Real-time PCR was performed to determine the relative mRNA expression of copper-zinc-superoxide dismutase (CuZnSOD, SOD1), glutathione peroxidase 1 (GPX1), catalase (CAT), NF-E2-related factor 2 (Nrf2), tumor necrosis factor-*α* (TNF*α*), interleukin-1*β* (IL-1*β*), IL-6, IL-8, and IL-10. *β*-Actin was used as a reference gene to normalize target gene transcript levels. Primers used in this study are shown in Table 2. The cDNA and primers were used to perform real-time PCR according to a previous study [16]. In brief, real-time PCR was performed in a total volume of 25 *μ*l, which consists of 1 *μ*g of cDNA template, 1 *μ*mol/l of forward and reverse primers, and 12.5 *μ*l SYBR Green Mix. The result was expressed as a ratio of the target gene to the reference gene using the formula 2^−(*ΔΔ*Ct)^.

### 2.6. Western Blot

Antibodies against NF-*κ*B (Bioss, China), p-NF-*κ*B (Bioss, China), and *β*-actin (Proteintech, USA) were used in this study. Briefly, frozen intestinal samples were homogenized and subsequently determined for protein concentration using the bicinchoninic acid (BCA) protein assay kit (Wellbio, China). Equal quantity of protein samples was separated on SDS-PAGE and then transferred to polyvinylidene difluoride (PVDF) membranes. After being blocked with 5% skim milk for 1 h at room temperature, the membranes were incubated with the indicated primary antibodies at 4°C overnight. Then, blots were stripped, followed by the incubation with HRP-conjugated secondary antibody (Proteintech, USA) for 1 h. The chemiluminescence signals were obtained using ECL Plus kits (Thermo, USA). The band density was normalized to *β*-actin and expressed as a relative level to control value.

### 2.7. Statistical Analysis

Data were analyzed by one-way ANOVA followed by Duncan's test using the SPSS statistical software. *P* < 0.05 was considered to be statistically significant. 0.05 < *P* < 0.10 was considered a tendency. All data are presented as mean ± SEM.

## 3. Results

### 3.1. Effect of Different Zn Sources on Growth Performance in Piglets under Oxidative Stress

The results of growth performance are presented in Table 3. During the 14 days of the prestarter period, dietary HMZn or ZnSO_4_ supplementations did not affect FCR or fecal sore in piglets (*P* > 0.05). Compared with the control group, diquat injection reduced ADG and increased FCR and fecal score (*P* < 0.05) during the 7 days of the starter period. HMZn and ZnSO_4_ both failed to exert notable protective effect on growth performance, while HMZn supplementation notably decreased fecal score.

### 3.2. Effect of Different Zn Sources on Intestinal Morphology in Piglets under Oxidative Stress

**Figure 1:**
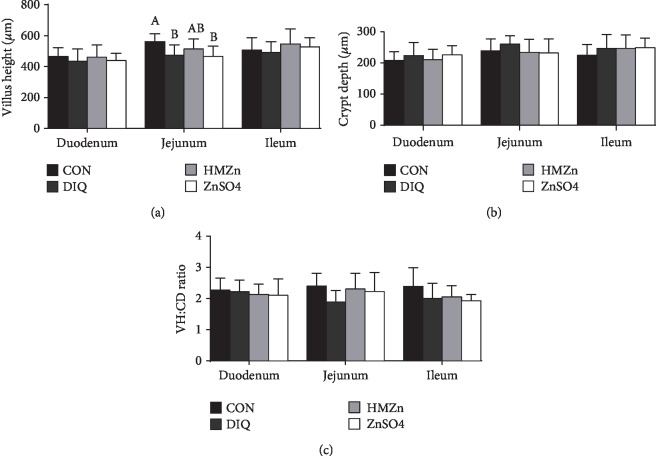
Effects of different dietary Zn sources on the small intestinal morphology in piglets under oxidative stress. Values are expressed as mean ± SEM, *n* = 6. ^a,b^Means of the bars with different letters were significantly different compared to the other groups (*P* < 0.05).

As shown in Figure 1, diquat exposure significantly decreased villus depth in the jejunum, while exerted no effect on the ratio of villi and crypt. Dietary HMZn supplementation tended to increase jejunal villus depth compared with NC groups (*P* > 0.05).

### 3.3. Effect of Different Zn Sources on Serum Antioxidant Capacity in Piglets under Oxidative Stress

**Figure 2:**
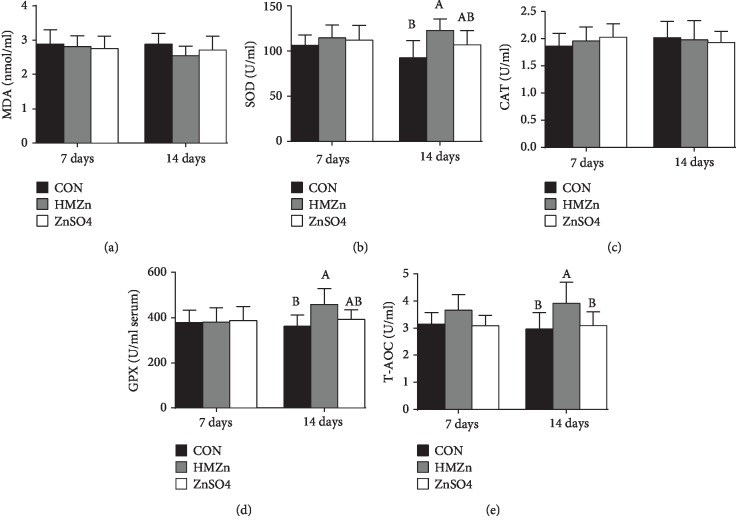
Effects of different dietary Zn sources on antioxidant capacity of piglets before diquat challenge. Values are expressed as mean ± SEM, *n* = 6. ^a,b^Means of the bars with different letters were significantly different compared to the other groups (*P* < 0.05).

**Figure 3:**
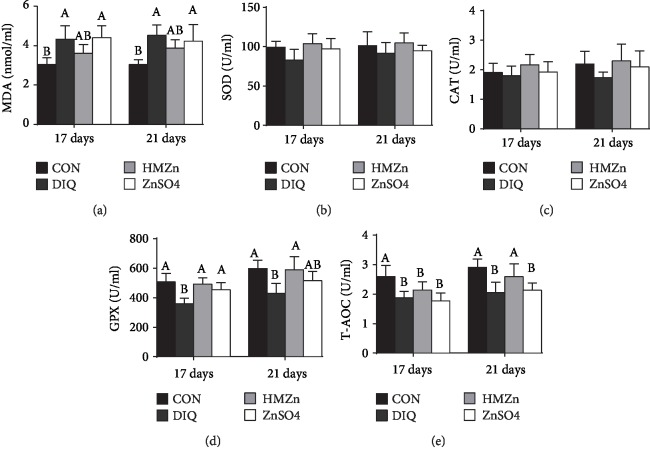
Effects of different dietary Zn sources on serum antioxidant capacity in piglets under oxidant stress. Values are expressed as mean ± SEM, *n* = 6. ^a,b^Means of the bars with different letters were significantly different compared to the other groups (*P* < 0.05).

**Figure 4:**
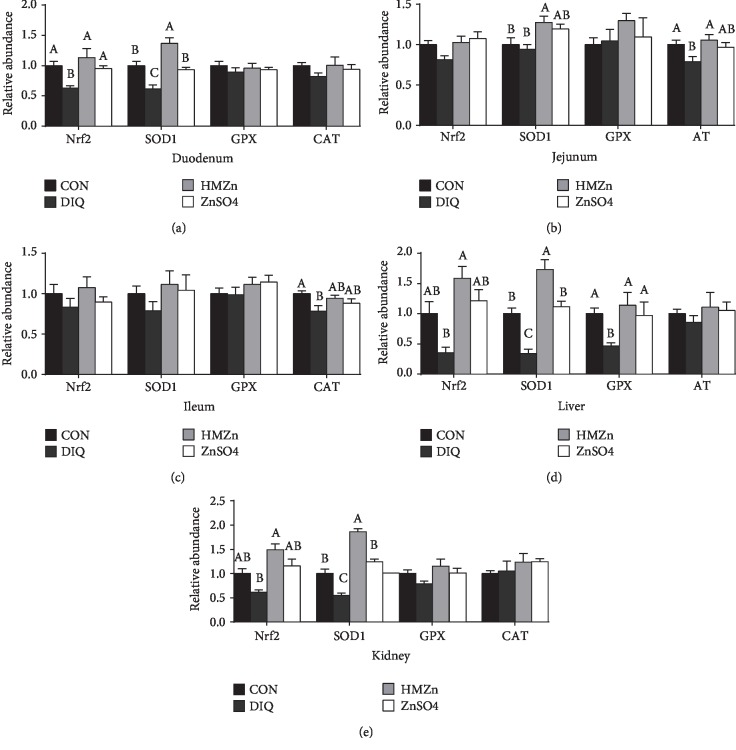
Effects of different dietary Zn sources on mRNA expression of antioxidant-related genes in piglets under oxidative stress. Values are expressed as mean ± SEM, *n* = 6. ^a,b^Means of the bars with different letters were significantly different compared to the other groups (*P* < 0.05).

As shown in Figure 2, serum MDA content was not affected by different sources of Zn supplementation during the prestarter period but was notably increased by diquat 3 days and 7 days after injection (*P* < 0.05), suggesting that the oxidative stress model was well established. Compared with the Con group, the activities of serum SOD, GPX, and T-AOC in the HMZn group were increased on day 14 (*P* < 0.05), while CAT remained unaffected. The serum antioxidant capacity of postinjection is illustrated in Figure 3. On day 17 (3 days post injection), the MDA content in serum was notably increased by diquat injection (*P* < 0.05), and the tendency was remained on day 21. The activities of serum GPX and T-AOC were notably decreased on days 17 and 21 (*P* < 0.05). It is noteworthy that both HMZn and ZnSO_4_ effectively attenuated the diquat-induced suppression of GPX activity. The relative mRNA expressions of antioxidant pathway-related genes in the small intestine, liver, and kidney are illustrated in Figure 4. Diquat exposure decreased the relative mRNA level of Nrf2 in the liver and CAT in the jejunum and ileum compared with the CON group (*P* < 0.05). While both HMZn and ZnSO_4_ showed notable effect on attenuating the diquat-induced suppression of mRNA expression of Nrf2, SOD1, and CAT, HMZn was proved to be more effective.

### 3.4. Effect of Different Zn Sources on Proinflammatory Cytokines in the Small Intestine of Piglets under Oxidative Stress

**Figure 5:**
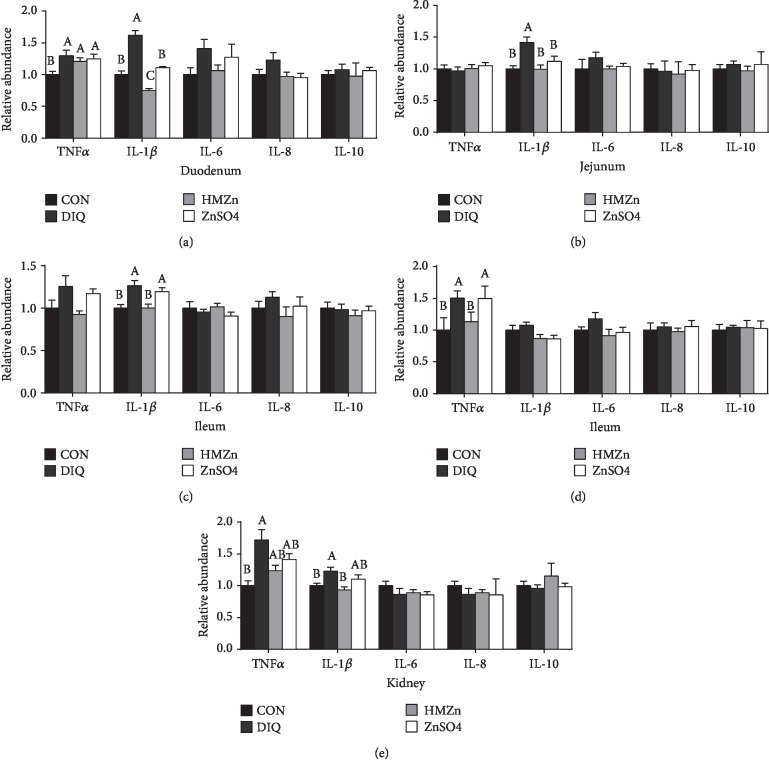
Effects of different dietary Zn sources on mRNA expression of proinflammatory cytokines in piglets under oxidative stress.

**Figure 6:**
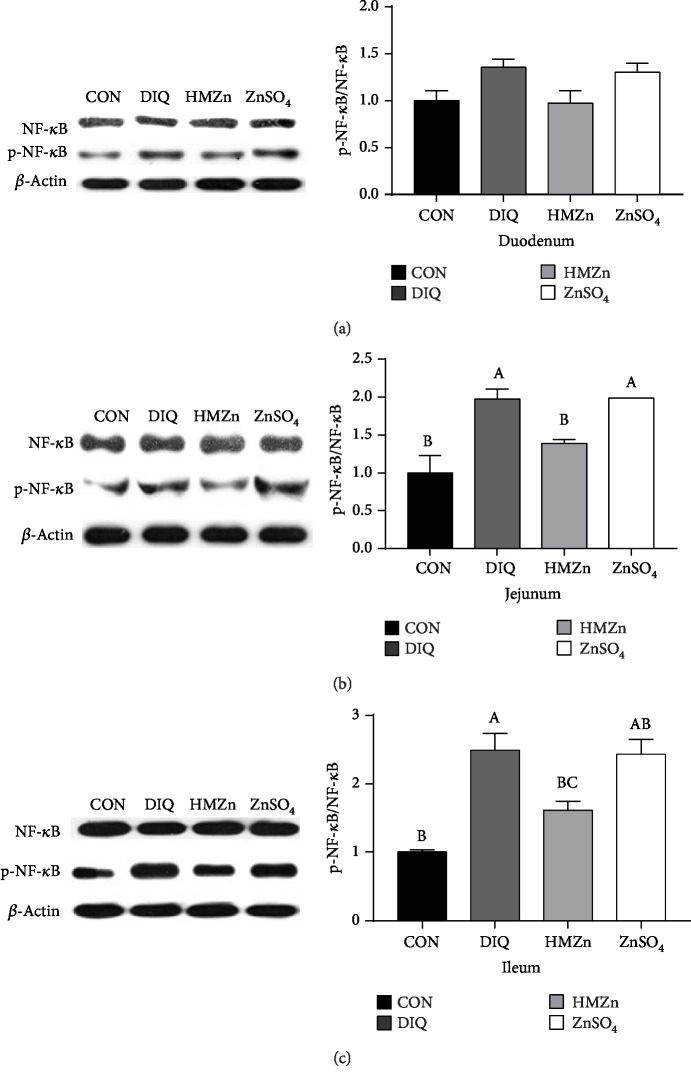
Effects of different dietary Zn sources on phosphorylation of NF-*κ*B in piglets under oxidative stress.

Diquat injection significantly increased the relative mRNA levels of TNF*α* and IL-1*β* compared with the control group (*P* < 0.05). Both HMZn and ZnSO_4_ supplementations alleviate the proinflammatory effect of diquat (Figure 5), and HMZn exhibited notable advantage over ZnSO_4_. Western blot results showed diquat challenge remarkably upregulated phosphorylation of NF-*κ*B protein (*P* < 0.05) (Figure 6). HMZn effectively lowered the NF-*κ*B phosphorylation while ZnSO_4_ showed little alleviating effect.

## 4. Discussion

Zinc, as a component of some antioxidant enzymes and proteins (i.e., SOD, glutathione) involved in the subsequent scavenge progress, participates in antioxidant defense by activating the activity of several enzymes [19]. Previous studies in humans and mice revealed that Zn deficiency from marginal to moderate led to growth retardation, immunity disorder, gastrointestinal impairment, and aggravated oxidative stress [20, 21].

In our study, the basal diet in the current experiment contained 80 mg/kg of Zn to meet the suggested NRC (2012) requirement. Zn supplementations did not have an impact on growth performance and diarrhea rate during the prestarter period. But after diquat injection, the effect of additional Zn supplementation on growth performance and diarrhea rate became significant. It can be explained that additional Zn supplementation facilitated the synthesis of Zn-containing antioxidant enzymes, thus improving the defense ability against oxygen-free radicals, which is helpful in maintaining the intestinal health and reducing inflammatory processes after diquat challenge, and finally improving growth performance. The data also suggested that HMZn had better effect than ZnSO_4_ on decreasing diarrhea, which is consistent with another study conducted in broiler chicks [22].

Villus height, crypt depth, and the ratio of villi and crypt are commonly used as important indicators for estimating intestinal integrity, which play critical roles in nutrient absorption [23]. Weaning stress often leads to villus impairment and malabsorption, finally resulting in diarrhea and retarded growth in weaned piglets [24]. Yin et al. (2015) reported that diquat challenge caused intestinal morphological injury [25]. Similarly, the present study showed that diquat challenge significantly decreased jejunal villus height but showed no negative effect on the rest of the recorded indices in intestinal morphology. Furthermore, villus height and the ratio of villi and crypt did not change in response to difference sources of Zn supplementation, except a compensatory increase in jejunal villus height in HMZn-supplemented pigs. These were supported by the findings that Zn enhanced the integrity of the intestinal epithelial barrier and promoted villus growth and intestinal absorption.

Malondialdehyde (MDA) is a metabolite produced by lipid peroxidation and widely used as an indicator of oxidative stress [9, 26, 27]. In the present study, regardless of sources, Zn supplementation showed no positive effect on serum MDA concentration during the prestarter period. The serum MDA concentration was then significantly increased during the postinjection period, indicating that the oxidative stress model induced by diquat was successful.

There is a dynamic equilibrium between the antioxidant capacity of the body and the production of reactive oxygen species (ROS); however, excessive ROS caused by oxidative stress may impair the antioxidant defense system, thus further resulting in cellular damage, death, and even tissue injury [28–30]. Enzymatic antioxidants, including SOD, CAT, and GPX, and nonenzymatic antioxidants can eliminate ROS or inhibit their generation and have important function on maintaining redox homeostasis [26]. Our results suggested that before diquat injection, HMZn supplementation improved the activity of serum SOD1, GPX, and T-AOC during the prestarter period. During the starter period (17 d, 21 d), the activity of GPX and T-AOC was significantly inhibited. It is evident that CAT and GPX provide vital antioxidant defenses against the excess of cellular ROS by decomposing hydrogen peroxide (H_2_O_2_) (Cui et al. 2016; Jarosz et al. 2017). Comparison of the different Zn treatments revealed that HMZn supplementation was efficacious in enhancing serum antioxidative enzyme activities and decreasing serum MDA concentration, while ZnSO_4_ showed little effects in decreasing serum MDA concentration. Nrf2 can bind to the promoter region of genes of endogenous antioxidant enzymes and facilitate the gene transcription of these enzymes [31]. Scientific evidence showed that Zn has the potential to activate the Nrf2 signaling pathway and Nrf2 target antioxidant enzyme genes and in turn to enhance the expression of several antioxidative and cytoprotective genes (i.e., SOD, GPX1, CAT, and Nrf2) in mice [32, 33]. Our study further showed that diquat challenge downregulated the relative mRNA expression of Nrf2 and its target antioxidant enzymes in the liver, kidney, and intestine.

The NF-*κ*B signaling pathway plays an important role in the regulation of inflammation, immune responses, and many cellular stress responses [26]. In the present study, phosphorylations of NF-*κ*B in the jejunum and ileum were significantly enhanced by diquat injection, as well as the elevation of relative mRNA expression of inflammatory factors in the duodenum, jejunum, ileum, liver, and kidney. HMZn supplementation exhibited notable alleviating effect on the diquat-induced inflammatory response, and the results suggested that HMZn may work through NF-*κ*B pathways.

## 5. Conclusion

Our results indicated that 80 mg/kg of Zn in the basal diet is not sufficient for adequate antioxidant enzyme activities in pigs, and addition of 200 mg/kg Zn was effective in protecting weanling piglet from oxidative stress by improving antioxidant systems. Dietary supplementation with 200 mg/kg Zn as HMZn provided more potent protection compared with the equal level of Zn as ZnSO_4_. However, more studies are required to explore long-term effects of HMZn in pigs and further investigate its underlying mechanisms of the antioxidant property.

## Figures and Tables

**Table 1:** Composition and nutrient level of the basal diets.

Items	Basal diet
Ingredient (%)	
Corn meal	60
Extruded soybean	10
Soybean meal	15
Fish powder	9.5
Whey powder	3
Premix^∗^	2.5
Composition	
Metabolism energy (MJ)	13.23
Crude protein (%)	18.95
Met (%)	0.27
Zn (mg/kg)	19.65

^∗^Premix (per kg of diet): vitamin A, 30 mg; cholecalciferol, 0.5 mg; vitamin C, 120 mg; vitamin E, 250 mg; menadione, 52 mg; vitamin B_1_, 18 mg; vitamin B_2_, 150 mg; vitamin B_6_, 5.5 mg; vitamin B_12_, 0.33 mg; nicotinic acid, 300 mg; folic acid, 4.2 mg; CuSO_4_·5H_2_O, 400 mg; MnSO_4_·H_2_O, 120 mg; Fe[C_2_H_4_O_2_N]HSO_4_, 595 mg; KI, 0.24 mg; Na_2_SeO_3_, 0.24 mg; CaHPO_4_, 1. 2 g.

**Table 2:** Primers used for quantitative reverse transcription-PCR.

Genes	Primers	Product length (bp)
*β-Actin*	F: CTGCGGCATCCACGAAACT	147
	R: AGGGCCGTGATCTCCTTCTG	

*SOD1*	F: TCCATGTCCATCAGTTTGGA	250
	R: CTGCCCAAGTCATCTGGTTT	

*GPX1*	F: CTTCGAGAAGTTCCTGGTGG	232
	R: CCTGGACATCAGGTGTTCCT	

*CAT*	F: CCACTAATGTCCAGCGTCT	159
	R: CAGCCTTATTCACCACTACCTG	

*Nrf2*	F: AGTGCAAGGCGGAGGTGA	235
	R: AGCCCGTTGGTGAACATAG	

*TNFα*	F: ACCACGCTCTTCTGCCT	128
	R:GGCTTATCTGAGGTTTG	

*IL-1β*	F: CAGCTGCAAATCTCTCACCA	85
	R: TCTTCATCGGCTTCTCCACT	

*IL-6*	F: TTCACCTCTCCGGACAAAC	122
	R: TCTGCCAGTACCTCCTTGCT	

*IL-8*	F: ACTTCCAAACTGGCTGTTGC	120
	R: GGAATGCGTATTTATGCACTGG	

*IL-10*	F: TAATGCCGAAGGCAGAGT	134
	R: GGCCTTGCTCTTGTTTTCAC	

**Table 3:** Effects of different dietary Zn sources on growth performance in diquat-challenged piglets.

Items	CON	DIQ	HMZn	ZnSO_4_	*P* value
Prestarter (0~14 days)					
ADG (kg/d)	0.23 ± 0.01	0.21 ± 0.02	0.23 ± 0.03	0.24 ± 0.02	0.931
ADFI (kg/d)	0.42 ± 0.01	0.39 ± 0.01	0.4 ± 0.03	0.45 ± 0.02	0.279
FCR	1.87 ± 0.09	1.96 ± 0.09	1.83 ± 0.13	1.93 ± 0.08	0.787
Fecal score	2.15 ± 0.2	2.05 ± 0.15	1.72 ± 0.13	1.79 ± 0.14	0.236
Starter (15~21 days)					
ADG (kg)	0.39 ± 0.05^a^	0.21 ± 0.03^b^	0.33 ± 0.03^ab^	0.27 ± 0.04^ab^	0.042
ADFI (kg)	0.84 ± 0.15	0.64 ± 0.11	0.79 ± 0.2	0.71 ± 0.13	0.805
FCR	2.15 ± 0.19^b^	3.05 ± 0.21^a^	2.42 ± 0.20^ab^	2.63 ± 0.23^ab^	0.049
Fecal score	2.36 ± 0.08^b^	3.54 ± 0.25^a^	2.55 ± 0.18^b^	3.18 ± 0.23^a^	<0.01

ADG = average daily gain; ADFI = average daily feed intake; FCR = food conversion rate. Values are expressed as mean ± SEM, *n* = 8. ^a,b^Means within a row with different letters were significantly different compared to the other groups (*P* < 0.05).

## Data Availability

The data used to support the findings of this study are available from the corresponding author upon request.
